# Implication of Oxidative Stress in Fetal Programming of Cardiovascular Disease

**DOI:** 10.3389/fphys.2018.00602

**Published:** 2018-05-23

**Authors:** Pilar Rodríguez-Rodríguez, David Ramiro-Cortijo, Cynthia G. Reyes-Hernández, Angel L. López de Pablo, M. Carmen González, Silvia M. Arribas

**Affiliations:** Departamento de Fisiología, Facultad de Medicina, Universidad Autónoma de Madrid, Madrid, Spain

**Keywords:** oxidative stress, fetal programming, cardiovascular diseases, fetal growth restriction (FGR), mitochondrial dysfunction

## Abstract

Lifestyle and genetic background are well known risk factors of cardiovascular disease (CVD). A third contributing factor is suboptimal fetal development, due to nutrient or oxygen deprivation, placental insufficiency, or exposure to toxic substances. The fetus adapts to adverse intrauterine conditions to ensure survival; the immediate consequence is low birth weight (LBW) and the long-term effect is an increased susceptibility to develop CVD in adult life. This process is known as Developmental Origins of Health and Disease (DOHaD) or fetal programming of CVD. The influence of fetal life for the future cardiovascular health of the individual has been evidenced by numerous epidemiologic studies in populations suffering from starvation during intrauterine life. Furthermore, experimental animal models have provided support and enabled exploring the underlying mechanisms. Oxidative stress seems to play a central role in fetal programming of CVD, both in the response of the feto-placental unit to the suboptimal intrauterine environment and in the alterations of physiologic systems of cardiovascular control, ultimately leading to disease. This review aims to summarize current knowledge on the alterations in oxidative balance in response to fetal stress factors covering two aspects. Firstly, the evidence from human studies of the implication of oxidative stress in LBW induced by suboptimal conditions during intrauterine life, emphasizing the role of the placenta. In the second part we summarize data on specific redox alterations in key cardiovascular control organs induced by exposure to known stress factors in experimental animals and discuss the emerging role of the mitochondria.

## Fetal Programming of Cardiovascular Diseases

### The Developmental Origins Hypothesis

Cardiovascular diseases (CVD) are one of the leading causes of morbidity and mortality worldwide. Despite pharmacologic interventions and efforts to create awareness on the importance of healthy lifestyles, CVD and associated risk factors – hypertension, diabetes, and obesity – remain among the most resilient health problems.

In addition to lifestyle factors -smoking, sedentarism, unhealthy diets- and the genetic background of the individual, decades of research have evidenced the key role of fetal and perinatal life on the development of CVD. This phenomenon is known as *fetal programming of disease* or *Developmental Origins of Health and Disease (DOHaD)*. The fetal programming hypothesis was proposed by Dr. Barker based on epidemiologic studies demonstrating the association between low birth weight (LBW), as a consequence of poor maternal nutrition, and development of coronary heart disease and hypertension in adult life (Forsdahl, 1977; Barker and Osmond, 1986; Barker and Osmond, 1988). The hypothesis has been widely validated by means of numerous epidemiologic studies in populations exposed to starvation in different parts of the world, which confirm the association between an adverse fetal environment and increased risk of hypertension, diabetes and obesity in adult life (Stein et al., 1996; Roseboom et al., 2000; Hult et al., 2010; Wu et al., 2017). The responses initiated *in utero* by undernutrition are also modulated during the perinatal period. Thus, it has been observed that an accelerated perinatal growth trajectory in individuals born with LBW has an additional negative impact, acting as a second hit, further contributing to CVD programming (Eriksson et al., 2000; Law et al., 2002; Singhal and Lucas, 2004; Leunissen et al., 2012).

### Fetal Stress Factors Implicated in DOHaD

Together with undernutrition, other stress factors that perturb the intrauterine environment have also been associated with inadequate fetal growth and subsequent development of CVD. One of these stress factors is fetal overnutrition – another form of malnutrition- due to Gestational Diabetes Mellitus (GDM), maternal obesity or excess weight gain during gestation. Obstetric complications also compromise intrauterine environment, particularly those related to placental insufficiency, like pre-eclampsia (PE), which reduce blood flow and nutrient and oxygen access to the fetus. A third factor interfering with fetal growth is exposure to environmental pollutants or toxic substances related to lifestyle, such as tobacco and alcohol. Fetal development is also affected by excess glucocorticoids, either due to maternal pharmacologic treatments or alterations in 11-β-Hydroxysteroid dehydrogenase (11-β-HSD), the placental enzymatic barrier limiting access of cortisol to the fetus.

**FIGURE 1:**
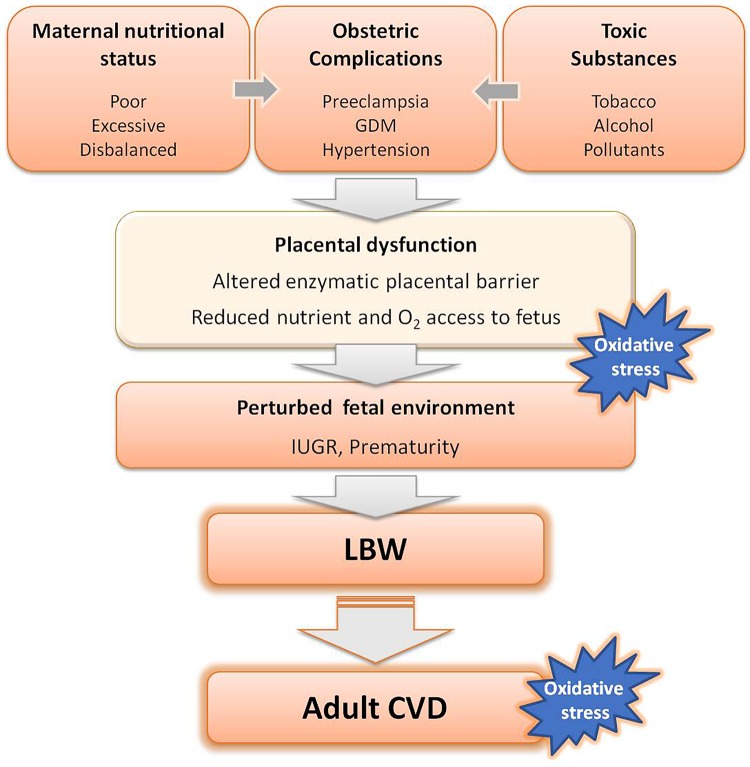
Adverse factors known to interfere with fetal growth leading to low birth weight (LBW) and subsequent development of cardiovascular diseases (CVD) in adult life. GDM, gestational diabetes mellitus.

Epidemiologic and experimental animal studies have demonstrated that all the above mentioned stress factors alter placental function. Thus, the placenta appears to play a central role in fetal programming, as the interface between the adverse intrauterine environment and the fetus (Alexander et al., 2015; Morton et al., 2016). A summary of the stress factors implicated in fetal programming is depicted in **Figure 1**.

Many of these stress factors are prevalent nowadays and fetal programming will likely contribute to the burden of cardiovascular and metabolic diseases in the next generations. Firstly, undernutrition is a chronic problem still prevalent in many parts of the world. Prematurity is another worldwide rising problem (Blencowe et al., 2013) which has been linked to CVD risk in early life. A recent study in a large cohort including over 2 million individuals born in Sweden from 1987 to 2012, found a strong association between preterm birth and incident heart failure in childhood and young adulthood (Carr et al., 2017).

Obstetric-related pathologies are also a global rising problem, particularly PE; in low-income countries associated with deficient diets and adolescent pregnancies and in high-income societies mainly due to the increase in maternity age (Ramiro-Cortijo et al., 2017). PE is associated with fetal growth restriction (FGR). Epidemiological evidence indicates that children born from pre-eclamptic mothers exhibit higher blood pressure levels (Paauw et al., 2017). FGR is also strongly associated with fetal cardiac and arterial remodeling and a subclinical state of cardiovascular dysfunction (Crispi et al., 2018) and there is also evidence of increased risk of stroke (Kajantie et al., 2009).

Maternal obesity, GDM and unbalanced diets are also widespread in high-income countries, leading to macrosomic neonates, also at risk of cardiometabolic programming. In a cohort longitudinal study, Boney et al. (2005) demonstrated that large for gestational age offspring of mothers with GDM or obesity were at significant risk of developing metabolic syndrome in childhood. It has also been shown that maternal obesity itself is another risk factor for early overweight (Gillman et al., 2003). These data suggest that the nutritional and metabolic environment of the mother may permanently program the offspring toward the development of metabolic syndrome (Xita and Tsatsoulis, 2010).

### Mechanisms Implicated in Fetal Programming of CVD

**FIGURE 2:**
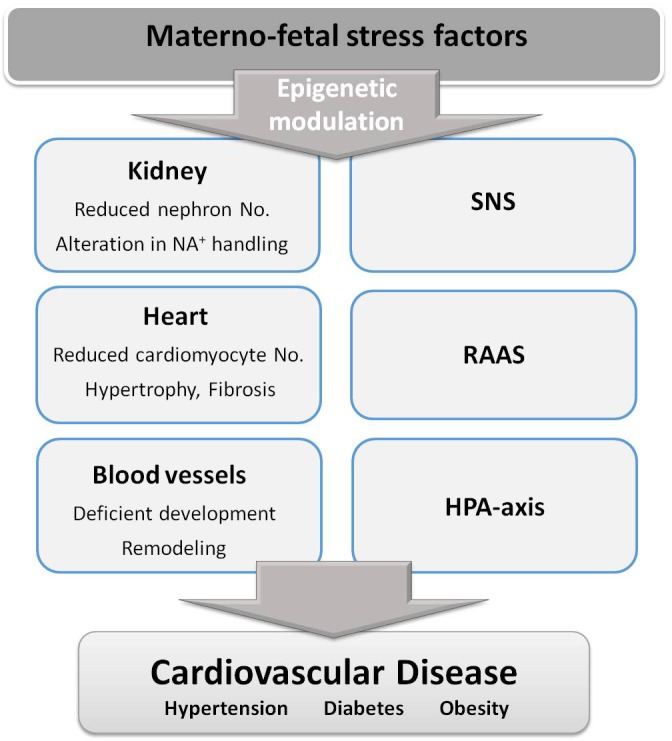
Stress factors during fetal life alter several organs and physiological systems, through epigenetic changes, contributing to cardiovascular disease development. SNS, sympathetic nervous system; RAAS, renin-angiotensin-aldosterone system; HPA-axis, hypothalamic-pituitary-adrenal axis.

Experimental animal studies have provided support to the DOHaD hypothesis and demonstrated the mechanisms implicated. They have also enabled exploring possible targets for therapeutic interventions and to conduct longitudinal studies to evaluate the effect of aging. It has been found that different fetal stress factors exhibit some common mechanisms leading to hypertension and heart disease, with slight differences in their relative implication depending on the stress factor. The following have been consistently demonstrated: (1) deficient kidney development leading to reduced nephron number and inadequate Na^+^ handling, (2) alterations in blood vessels, including remodeling, stiffening and endothelial dysfunction and (3) heart alterations, namely lower cardiomyocyte number followed by fibrosis and hypertrophy, leading to cardiac dysfunction. Animal studies have also consistently demonstrated the implication of the renin-angiotensin-aldosterone system (RAAS), the sympathetic nervous system, and the hypothalamic-pituitary-adrenal axis (HPA axis) in fetal programming of CVD. In addition, there is growing evidence that some of the above-mentioned alterations are mediated by epigenetic modulation of the expression of genes implicated in growth and cardiovascular control. A summary of the main mechanisms associated with fetal programming of CVD are shown in **Figure 2**. Animal studies have also evidenced a sexual dimorphism in fetal programming of CVD and demonstrated that females exhibit some protection, particularly against hypertension development. However, it is not established whether women are at lower risk than men and additional studies are needed to fully address the impact of sex on DOHaD (Alexander et al., 2014).

## Oxidative Stress and DOHaD

**FIGURE 3:**
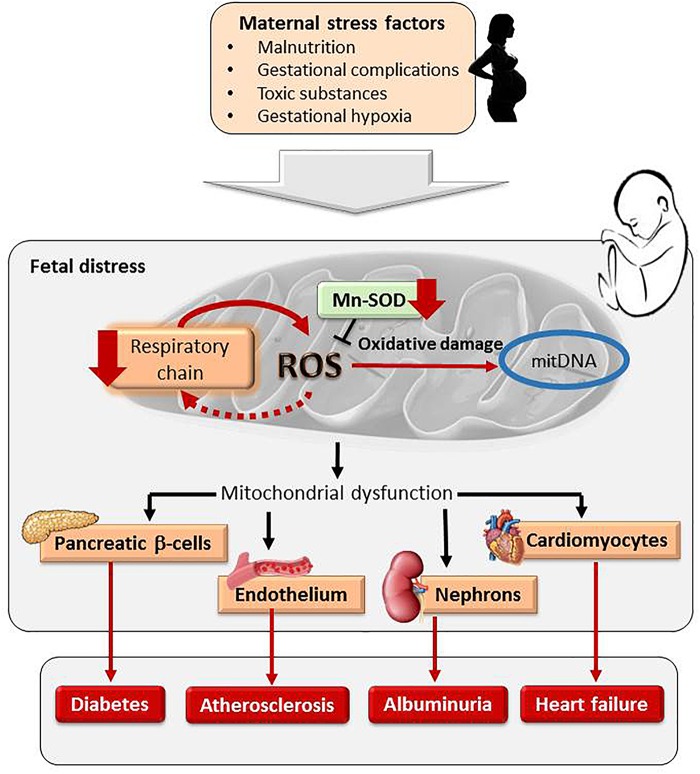
Maternal exposure to stress factors during pregnancy programs fetal mitochondria. An increase in ROS, respiratory chain inhibition and a vicious circle of ROS-induced ROS release and a reduction of mitochondrial antioxidant system MnSOD damages mitDNA. Mitochondrial programming in response to intrauterine stress factors has been found in pancreatic β-cells, cardiomyocytes, vascular endothelium and nephrons, contributing to cardiometabolic diseases.

Oxidative stress is the basis of many adult diseases, including CVD. Oxidative stress can be defined as an imbalance between the production and elimination of reactive species, in favor of the first, leading to oxidative damage to macromolecules. Relevant reactive species participating in oxidative stress in biological systems are reactive oxygen species (ROS) and nitrogen oxygen species (RNS). These substances are inevitably produced because of aerobic metabolism and are highly reactive, particularly free radicals. Therefore, biological systems have developed a network of antioxidants to maintain redox balance. The variety of antioxidants is very large, including hormones -such as melatonin- enzymatic systems and vitamins and other low molecular weight substances which can be endogenously produced or obtained from the diet. Their coordinated actions and specific location within cells and in biological fluids, enables keeping ROS and RNS at optimum concentrations to maintain a redox balance. However, an overproduction of ROS or a deficiency of antioxidants, lead to a pro-oxidative cellular state, which damages macromolecules. A summary of the redox balance is depicted in **Figure 3**. It is worth mentioning that ROS cannot be always considered “harmful substances,” neither can antioxidants be taken as “protective molecules.” ROS play numerous physiological roles in cell defense, metabolism, growth and differentiation (Valko et al., 2007). An excess of antioxidants may not always confer additional protection, since they can transform into pro-oxidant molecules, depending on the nature and stability of the antioxidant and the biochemical environment (Halliwell, 2013).

During pregnancy, ROS exert several important roles; they participate in placentation and serve as signaling molecules, inducing the transcription of several genes. Therefore, they are required to allow for the normal progression of embryonic and fetal development. It must be considered that the embryo and fetus have low antioxidant capacity. Therefore, an excess production in ROS during the intrauterine period would lead to a pro-oxidative state compromising fetal growth (Dennery, 2010). Indeed, numerous data suggest that oxidative stress is at the basis of obstetric and fetal complications associated with LBW. Furthermore, oxidative stress has also been proposed as the underlying mechanism between inadequate fetal development and programming of CVD at later stages of life. This review aims to cover these two aspects emphasizing the role of the placenta and redox alterations in key organs responsible for cardiovascular control.

### Association Between Suboptimal Fetal Conditions, Oxidative Stress and LBW

#### Oxidative Stress and Undernutrition

Pregnancy requires an increased intake of macro and micronutrients for maternal and fetal needs and maternal malnourishment is well proven to lead to LBW and adverse perinatal outcomes. In fact, most LBW infants are born to undernourished mothers (World Health Organization [WHO], 2014). Even if maternal diet has sufficient macronutrients, micronutrient deficiencies, particularly iron, are very common in many countries (Black et al., 2013). Many micronutrients are co-factors of antioxidant enzymatic systems or are antioxidants themselves. Therefore, in mothers suffering malnutrition antioxidant deficiency may generate a situation of oxidative stress. Intervention strategies in undernourished pregnant women demonstrate that micronutrient supplementation reduces the risk of LBW (Ota et al., 2015) and improves the metabolic health of children (Ekström et al., 2016). Undernutrition has an impact on the placenta, leading to deficient development or reducing the enzymatic barrier 11-β-HSD, allowing for excess cortisol reaching the fetus. The catabolic actions of cortisol are associated with increased ROS production. Placental alterations induced by undernutrition are also associated with oxidative stress. Thus, exposure to low protein diet during pregnancy induces an increase in oxidative stress biomarkers in the placenta and metabolic dysfunction in the offspring; alterations which were prevented by treatment with the antioxidant Resveratrol (Vega et al., 2016). In pregnant women with deficient diets, antioxidant supplementation also improved the metabolic health of children, but no differences in oxidative stress biomarkers were found, either in the mother or in the offspring (Ekström et al., 2016). Our revision of the literature indicates that the relationship between maternal undernutrition, maternal oxidative stress and offspring cardiometabolic outcome remains insufficiently explored in humans.

#### Oxidative Stress and Gestational Complications

Although the pathophysiology of PE remains incompletely understood, there is a general agreement that a deficient placental vasculature and oxidative stress play important roles. A poor development of placental vessels disrupts the normal pattern of blood flow, reducing nutrient access to the fetus and is a potential risk of ischemia/reperfusion, excess ROS production and injury (Burton, 2009; Burton et al., 2009). In fact, increased biomarkers of oxidative damage to lipids and proteins has been found in plasma (Bernardi et al., 2008) and in the placenta from pre-eclamptic women and in HELLP syndrome, also related to PE (Zusterzeel et al., 2001).

Pregnancy-related oxidative stress might also result from a poor maternal antioxidant status, insufficient to counteract the physiological ROS elevation. This is particularly important around weeks 10–12 of gestation, when maternal circulation is established and oxygen rises within the placenta. In fact, oxidative damage in pre-eclamptic women has been suggested to be the consequence of insufficient endogenous antioxidants (Zusterzeel et al., 2001; Llurba et al., 2004). We have evidence that poor maternal plasma antioxidant status in the first trimester of gestation is associated with the subsequent development of an obstetric complication (Ramiro-Cortijo et al., 2016). An important antioxidant molecule during pregnancy is melatonin, a hormone with pleiotropic actions (Chen et al., 2013), both as a direct free radical scavenger (Reiter et al., 2000), and stimulating the expression of enzymatic antioxidants (Rodriguez et al., 2004). The role of melatonin on the feto-placental unit is supported by the observed decrease in melatonin levels and in receptor expression in the placenta from women with PE (Gitto et al., 2004; Lanoix et al., 2012). In a cohort of healthy women at week 10 of pregnancy, we have found a negative correlation between plasma levels of melatonin and carbonyls, supporting the protective role of this hormone against protein oxidation (Ramiro-Cortijo et al., 2016). Recent animal studies in models of developmental programming have evidenced the potential implication of melatonin as a reprogramming strategy to prevent DOHaD-related diseases (Tain et al., 2017). Future human studies and clinical trials are needed to translate this information from animal models.

Maternal oxidative stress not only has an impact on maternal health, but may compromise fetal development, either directly or indirectly through reduction in placental perfusion and fetal nutrition (Karowicz-Bilinska et al., 2007; Hracsko et al., 2008). Increased biomarkers of oxidation have been found elevated in maternal and cord plasma from pregnant women carrying FGR fetuses (Biri et al., 2007; Kimura et al., 2013). Oxidative damage to the placenta has been proposed to be responsible for fetal growth restriction, as demonstrated by the higher placental levels of 8-OHdG -a marker of DNA oxidative damage- in pre-eclamptic pregnancies with FGR compared to those without fetal growth compromise (Fujimaki et al., 2011). Hypoxia may be implicated in these alterations as shown by the elevation in HIF-1 in the placenta from pre-eclamptic women together with higher levels of DNA oxidative damage and fetal growth restriction (Kimura et al., 2013).

Oxidative stress in LBW infants is also associated with CVD risk factors in early life. A close relationship between lipid peroxidation levels and cardiometabolic alterations -high blood pressure, altered lipid profiles and insulin resistance- has been found in children born FGR or small for gestational age (Franco et al., 2007; Mohn et al., 2007; Chiavaroli et al., 2009).

Premature infants are another group at risk of programming of CVD where oxidative stress has been shown to play a role. They are born with LBW before organogenesis is complete and they are commonly exposed to several stress factors known to be implicated in DOHaD, namely inadequate nutritional status or exposure to glucocorticoids. Epidemiologic studies reveal that preterm neonates exhibit hypertension in adolescence and as young adults (de Jong et al., 2012). Hypertension development in individuals born premature is associated to alterations in RAAS and sympathetic nervous system, as well as to renal and vascular abnormalities (Sutherland et al., 2014), i.e., through similar mechanisms to those found in fetal programming. Oxidative stress is also a strong contender in the association between prematurity and high blood pressure development. Preterm infants are especially vulnerable to ROS due to immaturity and insufficient antioxidants (Shim and Kim, 2013). Low levels of vitamins E and A and catalase have been reported in preterm infants, associated with increased levels of lipid and protein oxidation (Negi et al., 2012; Abdel Ghany et al., 2016). Moreover, preterm infants are exposed to catheters, mechanical ventilation, which may induce high levels of ROS. Thus, the imbalance between ROS production and antioxidant defenses may contribute not only to some common diseases of prematurity (Shim and Kim, 2013), but also to long tern development of cardiovascular and metabolic diseases.

#### Oxidative Stress in Response to Toxic Substances

The fetus is a potential target for toxics, including substances related to lifestyle factors, such as tobacco and alcohol, as well as environmental pollutants. These substances cross the placenta and are known to compromise development, particularly in the embryo. In addition, there is increasing evidence that they may also program the fetus for later development of CVD.

The negative effects of tobacco consumption during pregnancy on fetal development are well documented. Smoking is associated with preterm birth and FGR (Leonardi-Bee et al., 2008; Banderali et al., 2015). Even low cigarette consumption during pregnancy seems to have an effect on birth weight (Berlin et al., 2017). Furthermore, there is also evidence that smoking programs the offspring to hypertension, already observed in childhood, through endothelial dysfunction and renal alterations (Banderali et al., 2015). The mechanisms implicated in this association have been explored in experimental animals and suggest that tobacco may induce oxidative stress in the developing organs (Al-Gubory, 2016). Several components of tobacco may contribute. The first is CO, which can induce a hypoxic effect due to combination with hemoglobin (Banderali et al., 2015). CO has also been associated with alterations in the vasculature, leading to a reduction in blood flow and fetal nutrient compromise. For example, higher indices of CO in exhaled air are associated with increased resistance of umbilical arteries (Machado et al., 2011). Furthermore, smoking in pregnancy is linked to umbilical cord structural alterations and advance oxidation products (Rua Ede et al., 2014). Finally, CO might also compromise normal mitochondrial function due to its affinity to mitochondrial respiratory chain complex IV (Garrabou et al., 2016). Another substance in tobacco associated with fetal programming is nicotine, which exerts several negative effects in pregnancy, such as reducing uterine arterial blood flow (Al-Gubory, 2016) and promotes vascular oxidative stress and dysfunction in the offspring (Bruin et al., 2010). Nicotine replacement therapy has been developed as a pharmacotherapy for smoking cessation, but safety during pregnancy and the long-term effects on the progeny remains to be determined (Lim and Sobey, 2011).

Alcohol consumption during pregnancy has detrimental effects on placental formation and fetal growth (Kalisch-Smith and Moritz, 2017). In western countries, alcohol consumption is widespread in women of reproductive age, although they often stop drinking after discovering pregnancy. The first trimester appears to be the most sensitive period and there is also evidence that alcohol might have harmful effects even during the peri-conceptional period, increasing the risk of LBW and prematurity (Nykjaer et al., 2014). Alcohol effects are also linked to oxidative processes; ROS increases, depletion of antioxidants and elevation of biomarkers of oxidative damage have been reported in association with FGR (Nogales et al., 2017). Moreover, alcohol may lead to malnutrition and to selenium and folate deficiency (Ojeda et al., 2017), which are also linked to inadequate fetal development. These data point out the importance of population health policies, including educational programs to minimize tobacco and alcohol exposure, not only during pregnancy, but also during the peri-conceptional period.

While alcohol and tobacco can be voluntarily avoided, environmental pollutants are sometimes difficult or even impossible to evade. In large concentrations, these chemicals are teratogens. However, exposure to small doses during gestational periods might also interfere with fetal growth. Even if a single component might not induce an adverse effect, exposure to multiple contaminants has cumulative adverse outcomes, even at low concentrations (Al-Gubory, 2016).

Oxidative stress is also at the basis of the detrimental effects of fetal exposure to environmental toxics and their association with LBW and CVD programming. There is evidence of an inverse relationship between birth weight and concentration of pesticides in the placenta, maternal blood, cord blood, and breast milk (Lopez-Espinosa et al., 2007; Dewan et al., 2013). LBW also appears to be induced by exposure to polychlorinated biphenyls and heavy metals. Most environmental toxics induce abnormal ROS generation and oxidative stress, being a key target mitochondrial DNA oxidative damage (Al-Gubory, 2014, 2016). Other toxic substance is the halogen bromine, which is extensively used in industry. Experimental studies in mice have demonstrated that exposure to this oxidant gas during gestation induces blood pressure elevation in the dam, together with abnormal placental development. It was also found severe FGR, systemic inflammation and evidence of pulmonary and cardiac injury in the offspring. Since these are features similar to those found in PE, it has been suggested that it would be important to monitor pregnant women exposed to this toxic substance (Lambert et al., 2017).

Exposure to noxious substances during pregnancy are not only deleterious for cardiovascular function, but also program the fetus for respiratory diseases (Maritz et al., 2005; Petre et al., 2011; Harding and Maritz, 2012). In this sense, there is increasing evidence that chronic obstructive pulmonary disease (Stocks and Sonnappa, 2013) and asthma (De Luca et al., 2010) are associated with insults to the developing lung during fetal and early postnatal life when lung growth and development are rapid. Altered gene expression, through epigenetic modifications by environmental stress factors are among the most plausible mechanisms (Henderson and Warner, 2012; Jilling et al., 2017).

### Oxidative Stress and Mechanisms Implicated in Fetal Programming of CVD

#### Oxidative Stress-Induced Organ Damage

Oxidative damage in the kidney, blood vessels, and the heart are well known to contribute to organ dysfunction leading to CVD. Similar alterations have been found in animals exposed to different suboptimal intrauterine conditions, providing support for oxidative stress as a common underlying mechanism.

##### Kidney

Exposure to undernutrition, excess glucocorticoids, placental insufficiency or toxic substances during fetal life lead to alterations in nephrogenesis, Renin-Angiotensin-System (RAS), Na^+^ excretion and other renal alterations which contribute to hypertension (Paixão and Alexander, 2013). Alterations during the perinatal period are also important, particularly in species, such as rodents, where nephrogenesis continues after birth. Some of the kidney alterations are related to oxidative damage, as supported by the increase in renal markers of oxidative stress found in various animal models of developmental insult (Vehaskari et al., 2001; Stewart et al., 2005; Cambonie et al., 2007; Ojeda et al., 2007; Vieira-Filho et al., 2013). Some of the oxidative stress-related kidney alterations are linked to the RAAS dysfunction. Increased renal 8-isoprostane in response to Ang II (Bi et al., 2014), alterations in Ang II AT1/AT2 receptor subtypes and increased ROS-mediated Ang II responses are observed in association with hypertension development in animals exposed to fetal stress factors (Gwathmey et al., 2011).

##### Blood vessels

Oxidative stress is also a strong candidate contributing to the vascular alterations, particularly endothelial dysfunction, observed in animal models of fetal programming (Alexander et al., 2015; Morton et al., 2016). Both increased ROS/RNS production or defective antioxidants have been reported. There is evidence that increased superoxide generation, via activation of NADPH pathways, may contribute to vascular hyper-reactivity (Zhu et al., 2016). Vascular nitric oxide (NO) destruction by superoxide anion is one of the alterations consistently found in animals exposed to various fetal stress factors, namely nutrient deficiency, hypoxia, excess glucocorticoids, or placental insufficiency (Thompson and Al-Hasan, 2012). Excess superoxide anion interacts with NO, reducing its bioavailability and generating ONOO, a powerful oxidant which uncouples eNOS through oxidation of BH4, creating a vicious circle of superoxide anion generation (Morton et al., 2016).

Oxidative damage might also result from decreased antioxidants. We have evidence of a plasma deficiency in thiols, GSH, SOD, and melatonin, in pre-puberal rats exposed to fetal nutrient restriction (Rodríguez-Rodríguez et al., 2015). Plasma and tissue antioxidant deficiencies have also been found in goats exposed *in utero* to protein restriction (He et al., 2012) and SOD activity is reduced in the aorta of animals exposed to nicotine during fetal life (Xiao et al., 2011). The presence of oxidative damage prior to blood pressure elevation, supports a role of redox disbalance as a causative element implicated in fetal programming of hypertension (Rodríguez-Rodríguez et al., 2015).

##### Heart

Experimental animals of DOHaD provide evidence that hypoxia, nutrient deficiency or other stressors during intrauterine life, induce left ventricular hypertrophy (Gezmish et al., 2010; Gray et al., 2014; Zohdi et al., 2014; Rodríguez-Rodríguez et al., 2017). Animals exposed to fetal stress factors also exhibit a larger susceptibility to develop arrhythmias (Hu et al., 2000) and ischemia-reperfusion injury (Xu et al., 2006; Elmes et al., 2007).

The heart is the organ with the highest oxygen uptake and largest density of mitochondria and it is an active source of ROS. Therefore, the heart is a potential target of oxidative damage in situations of redox disbalance, such as those induced by stress factors during intrauterine life. Several lines of research support this hypothesis. Sheep exposed to dexamethasone *in utero* exhibit increased coronary artery ROS production (Roghair et al., 2008). Prenatal hypoxia also generates oxidative stress in fetal hearts in a variety of animal species (Thompson and Al-Hasan, 2012). Cardiac hypertrophy in response to nutrient restriction during fetal life is associated with increased heart NADPH oxidase expression prior to hypertension development (Rodríguez-Rodríguez et al., 2017) and oxidative and nitrosative damage (Rueda-Clausen et al., 2012; Tarry-Adkins et al., 2013).

Numerous experimental studies evidence a sexual-dimorphic response and a certain degree of protection in females, which do not develop hypertension or develop milder forms, (Alexander et al., 2014). Oxidative stress seems to contribute to the sex differences in fetal programming. For example, there is evidence of sex specific programming of the RAAS and subsequent ROS increase in association with hypertension development (Alexander et al., 2014). Antioxidant deficiency in animal models of fetal programming also seem to be sex-dependent. Thus, expression and activity of renal antioxidants are increased in female, but not in male offspring exposed to several fetal stress factors, namely placental insufficiency (Ojeda et al., 2013), betamethasone exposure (Bi et al., 2014) or undernutrition (Rodríguez-Rodríguez et al., 2015). A possible mechanism explaining the sexual dimorphism in fetal programming is the protective effects of estrogens, evidenced by the effects of castration or ovariectomy in experimental animal models (Alexander et al., 2014). Alternative mechanisms include a more adaptive growth strategies under stress conditions of females (Eriksson et al., 2000) or a better adaptation of the female placenta to suboptimal conditions (Rosenfeld, 2015).

#### Antioxidant Treatments

The implication of oxidative stress in fetal programming is also evidenced by the prevention of hypertension and organ damage by prenatal antioxidant treatment. Thus, increased Ang II-mediated contractile response in offspring from rats exposed to protein restriction during gestation is abolished by treatment of the dams with Lazaroid, a lipid peroxidation inhibitor, which also prevented the rise in blood pressure (Cambonie et al., 2007). Vitamin C and E treatments reversed the increased vasoconstrictor responses observed in animals exposed to postnatal glucocorticoids (Herrera et al., 2010) or to fetal hypoxia (Thakor et al., 2010). Similar effects are also observed with Allopurinol, a xanthine oxidase inhibitor (Kane et al., 2014). Antioxidant treatments also prevent renal or cardiovascular damage in response to placental insufficiency (Silva et al., 2011), prenatal glucocorticoid exposure (Roghair et al., 2011) or various models of undernutrition during fetal life (Elmes et al., 2007; Rexhaj et al., 2011; Vieira-Filho et al., 2011).

The above mentioned findings in animal models have encouraged clinical trials exploring the possible benefits of antioxidant therapy in compromised pregnancies. Unfortunately, results have not been as promising as expected. Some have failed to demonstrate improved pregnancy or fetal outcomes and, worryingly, several have demonstrated increased complications (Morton et al., 2016). This antioxidant paradox has been accounted for by Halliwell (2013) by means of various facts. On the one hand, laboratory animals seem more responsive to dietary antioxidants than humans. Secondly, some antioxidants (e.g., polyphenols and ascorbate) could exert mild pro-oxidant effects under some circumstances, related to the presence of transition metals. Thirdly, many clinical trials with antioxidants have not taken into consideration the baseline nutritional status of the population (Halliwell, 2013). In fact, this could account for the beneficial effects of supplements in undernourished populations, but not in those with normal nutritional status. It has also been proposed that mild pro-oxidant effects could even be beneficial, perhaps by increasing the levels of antioxidant defenses (Halliwell, 2013). In this sense moderate physical training could represent an alternative to pharmacological treatments. It is well known that moderate exercise appears to be beneficial for health, in part due to ROS-induced antioxidant defense systems (Pingitore et al., 2015). These aspects clearly warrant further investigation.

#### Mitochondria Programming

The role of the mitochondria in fetal programming is gaining attention. In hostile fetal environments -such as in situations of limited access to nutrients and oxygen- “mitochondria programming” might be a strategy for cell survival, but with later detrimental effects. For example, fetal hypoxia (induced by placental insufficiency or in high altitude pregnancies) has been shown to down-regulate oxidative metabolism and switch it to glycolytic pathways, along with mitochondrial dysfunction. These alterations have been suggested to participate in poor fetal growth leading to LBW, which is frequently found in pre-eclampsia and gestations at high altitude (Murray, 2012).

Mitochondria are the powerhouse that provides energy for cell function; therefore, a reduction in number or a functional alteration are likely to be detrimental for cells, particularly for those with high energy requirements such as pancreatic β-cells or cardiomyocytes. Alteration in β-cell mitochondrial function has been observed in animal models of undernutrition or placental insufficiency and has been proposed to explain the development of diabetes in later life (Simmons et al., 2005; Reusens et al., 2011). Similarly, a decrease in mitochondrial energetics has been found in skeletal and cardiac muscles of adult offspring from undernourished mothers and suggested to represent a compensatory mechanism programmed *in utero* to handle limited nutrient availability (Beauchamp and Harper, 2016). Decreased mitochondrial respiration rates are also a characteristic of heart failure. Therefore, it is possible that mitochondrial programming might provide the basis for the cardiac dysfunction observed in animal models of DOHaD (Rueda-Clausen et al., 2008; Rodríguez-Rodríguez et al., 2017).

Oxidative stress may contribute to mitochondrial dysfunction. During cellular respiration ROS are inevitably produced, but redox balance is maintained by elimination via Mn-SOD. If electrons leaking into the mitochondrial space are in excess the antioxidant system cannot eliminate the ROS overload. This situation might occur if oxidative phosphorylation is inhibited or if MnSOD is reduced. An excess ROS has deleterious consequences for the mitochondria. ROS inhibit electron transport chain activity leading to a vicious circle of ROS-induced ROS release (Zorov et al., 2006). On the other hand, ROS damage mitochondrial DNA (mitDNA), which has a limited repair system and lacks protective histone proteins (Reusens et al., 2011). Evidence of alterations in mitDNA has been reported in human and animal models of fetal programming. In this sense, umbilical cords from small for gestational age babies have lower mitDNA content (Sookoian et al., 2013). Alterations in mitDNA from β-cells have also been found in animal models of placental insufficiency and maternal undernutrition, in association with later development of diabetes (Simmons et al., 2005; Reusens et al., 2011). Mitochondria dysfunction in endothelial cells has also been proposed to be implicated in fetal programming of atherosclerosis (Leduc et al., 2010). There is also substantial evidence from human studies and animal models that environmental contaminants, alcohol and tobacco also induce mitDNA damage via ROS (Al-Gubory, 2014). For example, tobacco exposure during gestation in mice leads increased renal levels of mitochondrial-derived ROS in the progeny, a reduction in oxidative phosphorylation proteins and in the activity of mitochondrial MnSOD. These alterations preceded the onset of albuminuria (Stangenberg et al., 2015). A summary of the relationship between maternal stress factors, mitochondrial programming and cardiometabolic disease is shown in **Figure 3**.

The above-mentioned evidence supports the hypothesis that mitochondrial damage (“programming”) induced by suboptimal conditions during intrauterine life might provide the background for subsequent development of cardiometabolic diseases. However, there are many aspects still unresolved. Firstly, mitochondria are not homogeneous in cells and their response might differ depending on the type of stress factor, the tissue implicated and the time of insult. In addition, mitochondria are highly dynamic organelles and their activity can be regulated by fusion and migration (Chan, 2006). It has been found that mitochondrial dynamics can influence the response of the organelle to oxidative stress (Park and Choi, 2012). Furthermore, the response of mitochondria to fetal stressors appears to be modulated by sex, an important aspect in fetal programming which deserves further attention.

#### Epigenetic Modulation and Cell Senescence

Epigenetic modifications, i.e., heritable changes in gene expression that do not result from alterations in the nucleotide sequence, regulate cellular differentiation and fetal development. A mounting body of evidence implicates epigenetic modulation -DNA methylation, histone acetylation, or microRNA- as fundamental mechanism implicated in DOHaD and in the transmission of alterations to future generations.

The genes involved are starting to be revealed. Among others, RAS and NO pathways have been found altered in humans born LBW and in animal models of fetal programming. Hypomethylation of AT1b receptor has been evidenced in rodents exposed to fetal undernutrition (Bogdarina et al., 2007). Similarly, hypomethylation of the Angiotensin Converting Enzyme promoter was found in children born small for gestational age and related to blood pressure (Rangel et al., 2014). On the other hand, hypomethylation of eNOS promoter has been found in human umbilical endothelium from FGR fetuses (Krause et al., 2013).

Alterations in histone deacetylation processes are also implicated in fetal programming (Anwar et al., 2016). In this regard, sirtuins (SIRTs) may play a key role. SIRTs are a family of deacetylases and key regulators of vascular homeostasis. The most extensively studied is SIRT1, which regulate, among other processes, inflammation, endothelial function, mitochondrial biogenesis and aging. Oxidative stress might be a key player, since it reduces the histone deacetylation activity of SIRT1 (Hwang et al., 2013).

SIRT1 also preserves the normal telomere length and therefore, changes in its activity can be implicated in cellular senescence. Several common risk factors for CVD such as smoking, diabetes mellitus, hypertension, obesity and alcohol consumption, have been associated with short telomere length (Yeh and Wang, 2016). However, causality remains undetermined (Fyhrquist et al., 2013). Tissue inflammation and oxidative stress have been proposed as potential mechanisms implicated (Yeh and Wang, 2016; Vasconcelos-Moreno et al., 2017). Cellular senescence may also be programmed, as evidenced by several studies which have found an association between adverse intrauterine environment and shorter telomere length later in life (Entringer et al., 2011; Hallows et al., 2012; Tarry-Adkins and Ozanne, 2014), also observed in neonates with abnormal fetal growth (Tellechea et al., 2015). Among possible mechanisms inflammation, oxidative stress and energy failure due to mitochondrial dysfunction have been proposed (Von Zglinicki, 2002; de Almeida et al., 2017).

Epigenetic dysregulation induced by stress factors during intrauterine life, not only lead to CVD in the adult, but are also transferable to the next generation (Patel and Srinivasan, 2002). Fortunately, epigenetic modifications are reversible and interventions which modify epigenetic programming may represent an important and novel approach to reduce the burden of CVD (Anwar et al., 2016).

## Summary and Conclusions

Environmental stress factors during fetal and perinatal life can shape the future health of the individual and increase susceptibility to several adult diseases. Suboptimal intrauterine conditions induce alterations in placental redox balance, associated with poor fetal development. Furthermore, oxidative stress is also at the basis of changes in the kidney, heart, blood vessels, and cardiovascular control systems, ultimately leading to disease. There are some disparities, such as time of initiation of redox alteration, the implication of different antioxidants and reactive species or the organs which are more susceptible to oxidative damage. These differences may depend on the type of fetal stress factor, vulnerability of the gestational period, or age of study of the cardiovascular damage. In addition, sex appears to play an important role in the susceptibility to fetal programming, but the mechanisms behind it and the implication of oxidative stress are still not sufficiently explored. Therefore, additional research is needed to gain insight into how specific stress factors throughout pregnancy and perinatal life modulate organ growth, to develop specific strategies to reduce their impact on disease.

Another important aspect is that obstetric and fetal complications, malnutrition and exposure to toxic substances -well known fetal stress factors- are growing problems worldwide. Therefore, it is foreseen that fetal programming will contribute to the burden of CVDs in future generations. Epigenetic modulation of key genes implicated in cardiometabolic control seem to be at the origin of fetal programming. Therefore, interventions which modulate gene expression may represent a possible approach to counteract the deleterious effects of adverse intrauterine environment. In addition to the development of therapeutic interventions, other possible strategies are implementation of governmental policies aimed at reducing malnutrition or environmental pollutants and at promoting healthy lifestyles during gestation by means of education.

## Author Contributions

PR-R, DR-C, and CR-H searched and organized the data. PR-R, DR-C, CR-H, ALdP, MG, and SA wrote a section of first version of the manuscript. SA, MG, and ALdP wrote the final version. All authors contributed to manuscript revision, read and approved the submitted version.

## Conflict of Interest Statement

The authors declare that the research was conducted in the absence of any commercial or financial relationships that could be construed as a potential conflict of interest.
